# Vestibular and Ocular Motor Properties in Lateral Medullary Stroke Critically Depend on the Level of the Medullary Lesion

**DOI:** 10.3389/fneur.2020.00390

**Published:** 2020-06-05

**Authors:** Seung-Han Lee, Jae-Myung Kim, Bernhard Schuknecht, Alexander Andrea Tarnutzer

**Affiliations:** ^1^Department of Neurology, Chonnam National University Hospital and Chonnam National University Medical School, Gwangju, South Korea; ^2^Medical Radiological Institute, Zurich, Switzerland; ^3^Faculty of Medicine, University of Zurich, Zurich, Switzerland; ^4^Neurology, Cantonal Hospital of Baden, Baden, Switzerland

**Keywords:** video head impulse test, head-shaking nystagmus, gaze-evoked nystagmus, HINTS, Wallenberg syndrome

## Abstract

**Background:** Lateral medullary stroke (LMS) results in a characteristic pattern of brainstem signs including ocular motor and vestibular deficits. Thus, an impaired angular vestibulo-ocular reflex (aVOR) may be found if the vestibular nuclei are affected.

**Objective:** We aimed to characterize the frequency and pattern of vestibular and ocular-motor deficits in patients with LMS.

**Methods:** Patients with MR-confirmed acute/subacute unilateral LMS from a stroke registry were included and a bedside neuro-otological examination was performed. Video-oculography and video-based head-impulse testing (vHIT) was obtained and semicircular canal function was determined. The lesion location/extension as seen on MRI was rated and involvement of the vestibular nuclei was judged.

**Results:** Seventeen patients with LMS (age = 59.4 ± 14.3 years) were included. All patients had positive H.I.N.T.S. vHIT showed mild-to-moderate aVOR impairments in three patients (ipsilesional = 1; ipsilesional and contralesional = 1; contralesional = 1). Spontaneous nystagmus (*n* = 10/15 patients) was more often beating contralesionally than ipsilesionally (6 vs. 3) and was accompanied by upbeat nystagmus in four patients. Head-shaking nystagmus was noted in seven subjects, ipsilesionally beating in six and down-beating in one. On brain MRI, damage of the most caudal parts of the medial and/or inferior vestibular nucleus was noted in 13 patients. Only those two patients with lesions affecting the rostral medulla oblongata demonstrated an ipsilaterally impaired aVOR.

**Conclusions:** While subtle ocular motor signs pointed to damage of the central–vestibular pathways in all 17 patients, aVOR deficits were infrequent, restricted to those patients with rostral medullary lesions and, if present, mild to moderate only. This can be explained by lesions located too far caudally and too far ventrally to substantially affect the vestibular nuclei.

## Introduction

The human medulla oblongata is the most caudal brainstem area and is critically involved in various motor, sensory, and autonomic functions. Following the vascular supply, two distinct clinical syndromes may be distinguished in case of ischemic lesions, being restricted either to the paramedian ventral parts of the medulla (“Dejerine syndrome”) (1) or to the (dorso-)lateral aspects of the medulla oblongata [“Wallenberg syndrome” or lateral medullary stroke (LMS)] (2–4). The dorsolateral medullary area is supplied either by the posterior branches of the posterior inferior cerebellar artery or by the lateral branches of the vertebral artery itself (5). Thus, it is not surprising that vertebral artery dissection was found to be the most common cause of LMS (15–29%) (6).

Sensory input originating from the vestibular end organs (i.e., the semicircular canals and the otolith organs) is forwarded to the brainstem vestibular nuclei (VN) located in the dorsolateral area of the medulla oblongata for further processing and integration (7). Thus, in case of damage to the dorsolateral medulla oblongata, among other symptoms such as dissociated sensory loss, dysphagia, Horner syndrome, and hemiataxia, new-onset persistent vertigo or dizziness may be reported. On clinical examination, subtle ocular motor deficits including spontaneous nystagmus (SN), gaze-evoked nystagmus (GEN), and a deficient angular vestibulo-ocular reflex [aVOR, as assessed by the bedside horizontal head-impulse test (8)] may be observed. Patients with LMS often show saccadic lateropulsion, i.e., an asymmetry of saccadic amplitudes (with saccades being hypometric and requiring a series of saccades when looking away from the lesioned side) despite full movement range (9). While detecting subtle deficits of the brainstem, vestibular circuits may be challenging, and since saccadic lateropulsion may limit significantly the interpretation of the bedside head-impulse test, we asked to which extent the use of quantitative, video-based head-impulse testing (vHIT) may facilitate the detection of a deficient aVOR and thus further support damage to the most caudal parts of the brainstem. With the introduction of commercially available video goggles for quantifying the function of all six semicircular canals (10), fast and detailed assessment of the vestibular end organ became available recently.

We hypothesized that deficits in the aVOR are linked to damage of the VN as identified on structural MR imaging. Considering the anatomical distribution of the VN, the inferior and the medial VN are located in the dorsal and dorsolateral aspects of the medulla oblongata, whereas the superior and the lateral VN are situated further rostrally (i.e., in the pons) (11). Thus, we predicted an ipsilaterally impaired vHIT in those patients with damage of the dorsolateral portions of the medulla and compared the frequency of abnormal vHIT with other subtle ocular motor signs in these patients including SN, GEN, and head-shaking nystagmus (HSN) to gain further insights into which bedside and quantitative vestibular and ocular motor tests are most helpful in the assessment of LMS patients.

## Materials and Methods

### Patient Selection

This study was carried out in accordance with the recommendations of the Institutional Review Board of the Chonnam National University Hospital (Gwangju, South Korea) with written informed consent from all subjects. All subjects gave written informed consent in accordance with the Declaration of Helsinki. The protocol was approved by the Institutional Review Board of the Chonnam National University Hospital (Gwangju, South Korea).

We reviewed the Hospital's prospective stroke registry (time period: January 2014 and March 2018) for patients with MRI-confirmed LMS that either presented to the emergency department or to the outpatient clinic of the Department of Neurology, Chonnam National University Hospital, Gwangju, South Korea. Fifty-five consecutive patients were identified in this time period. Thirty-eight patients had to be excluded because of no agreement to perform the vHIT (*n* = 12), poor associated medical condition (*n* = 8; e.g., due to angina pectoris, pneumonia, and delirium), lack of dizziness or vertigo and having other symptoms such as sensory symptoms (*n* = 8), no pure brainstem infarction [e.g., combined cerebellar infarction (except lesions restricted to the flocculus/paraflocculus), *n* = 4], incomplete data (missing vHIT, *n* = 3), or other non-specified causes (*n* = 3). Eventually, 17 patients with LMS with typical neurologic (i.e., crossed sensory loss and/or ipsilateral Horner syndrome and/or truncal ataxia, etc.) and radiologic [acute infarction on MRI including diffusion-weighted imaging (DWI)] findings were included.

All patients underwent a structured bedside neuro-otologic examination in the emergency department of the Chonnam National University Hospital. This included an assessment of postural stability reporting the grade of truncal ataxia, as previously described by Moon and colleagues (12). Whereas grade 0 referred to preserved postural stability (i.e., being able to stand on Tandem Romberg with the eyes open for at least 3 s), truncal ataxia was rated as “mild” (=grade 1) if patients were unable to stand on Tandem Romberg with the eyes open for at least 3 s and as “moderate” (=grade 2) if patients were unable to stand on Romberg test with the eyes open for at least for 3 s. “Severe” truncal ataxia (=grade 3) was defined as the patient being unable to stand or sit without support.

### Video-oculography Recordings

Video-oculography (VOG; SLMed, Seoul, Korea) was performed in a sitting position for the detection of spontaneous (horizontal, vertical, or torsional) nystagmus, HSN, and GEN.

Horizontal head shaking was performed for 15 s with a frequency of about 2–3 Hz and an amplitude of ±10°. A central pattern of HSN was considered if HSN was beating ipsilesionally or if a perverted HSN was present (defined as vertical and/or torsional nystagmus developing in response to horizontal head shaking). If a vertical HSN was accompanied by a horizontal one, this was considered a perverted HSN only when the slow-phase velocity of the vertical component was at least 20% of that of the horizontal one (13). We used previously published cutoff values from healthy normal subjects to determine whether HSN was significant in velocity (cutoff values: horizontal HSN ≥3°/s; vertical HSN ≥2°/s, torsional HSN ≥2°/s) and duration (i.e., lasting more than 5 s) (14). Beforehand, the slow-phase velocity of any SN was subtracted as proposed by Choi and colleagues (14). Horizontal GEN was induced by horizontal (±30°) target displacement as previously described (15). GEN was only considered to be present when the recorded nystagmus was beating in the direction of gaze on both sides.

### Video-Head-Impulse Testing (vHIT)

Quantitative head-impulse testing was performed using a lightweight, portable VOG device (ICS Impulse; Otometrics, Taastrup, Denmark). Patients were sitting and asked to fixate a target at about 1.5 m distance. After eye-position calibration, the examiner performed a series of horizontal head impulses toward each ear. Vertical head impulses were applied to all subjects along the left anterior–right posterior (LARP) and right anterior–left posterior (RALP) canal plane. Target head velocity was between 150 and 200°/s and head displacement ranged between 10 and 20°. The pre-specified target number of head impulses per semicircular canal was 20.

OtosuiteV 4.0 (Otometrics) was used for analysis (gains, catch-up saccades) of the vHIT recordings. The aVOR gain was calculated as the ratio of cumulative slow-phase eye velocity over cumulative head velocity from the onset of the head impulse to the moment when head velocity returned to zero (10). Each compensatory saccade was visualized to ensure accurate characterization. Overt saccades were defined as compensatory saccades in the opposite direction of the head rotation that reached peak acceleration after the head had stopped moving. Covert saccades occurred in the opposite direction of the head rotation and reached peak acceleration before the head had stopped moving (16). Traces with artifacts (e.g., blinks during the vHIT) were removed interactively (17). If SN was present, OtosuiteV 4.0 was operated in the nystagmus-adjusted interpretation mode, which alters filtering algorithms for determining inadequate impulses and calculates aVOR-gain measures by accounting for the spontaneous, slow-phase drift of the eye. Built-in quantitative analysis of saccades (OtosuiteV 4.0) including classification of saccades [being either covert saccades (CS) or overt saccades (OS)], saccade peak velocity (°/s), and saccade latency (ms) was performed in all patients.

All vHIT traces were independently reviewed by two neuro-otologists with extensive experience (SHL, AAT) without knowledge of the clinical findings and the results from MR imaging. We adhered to the cutoff values in aVOR gain for the horizontal (0.8) and the vertical (0.7) canals proposed by the manufacturer of the video goggles used (Otometrics), which were in agreement with normative values for a wide range of ages reported (18). All traces were assessed by the two reviewers for (1) reduced aVOR gain and (2) increased corrective saccades or (3) a combination of both (19). Inter-rater agreement for individual canal function (normal vs. pathological) in all 17 patients included was 0.70 (Cohen's kappa) (20). Discordant ratings were resolved by discussion among the two reviewers.

### Neuroimaging

Following the diagnostic procedure of the Chonnam National University Hospital for patients suspected to have acute stroke, emergency MR imaging immediately after hospital admission was obtained in all patients. The MRI protocol consisted of DWI (slice thickness = 4 mm), FLAIR, gradient-echo imaging, and time-of-flight (TOF) MR angiography in a sequential manner.

Three axial sections of the medulla oblongata were identified (lower, middle, and upper level) according to Bradley (21) and were further divided into four different sections based on the vascular supply (anteromedial, anterolateral, lateral, and dorsal) (22, 23). The lower level was set at the termination of the fourth ventricle (with the medulla being relatively round shaped and without bulging of the lateral surface), the middle level was placed at the center of the inferior olivary nucleus (ION) (with resulting bulging of the lateral surface), and the upper level was set at the ponto-bulbar junction (with massive bulging of the dorsolateral area due to the restiform body) (2, 21).

MR sequences were reviewed by a board-certified neuroradiologist (BS) who was blinded to the clinical data. Specifically, the involvement of several predefined structures was assessed, including the medial VN, the lateral VN, the superior VN and the inferior VN, the nucleus prepositus (NP), the inferior cerebellar peduncle (ICP), the cerebellar vermis, the cerebellar hemispheres, and the ION. Note that no volumetric analysis was performed as the MR images available were obtained for clinical routine and not for research purposes.

## Results

### Demographics and Neuro-Otologic Findings

All 17 patients included [aged 59.4 ± 14.3 years (average ± 1 standard deviation); 8 females] reported acute-onset dizziness or vertigo, whereas one patient in addition also noticed diplopia (see Table 1 for further details). While the majority of patients presented to the emergency department within 24 h of symptom onset (10/17, 59%), delay was up to 16 days in the others. Frequent clinical findings on neurological examination included ipsilesional facial hypesthesia (12/17), contralesional body hypesthesia (10/17), ipsilesional Horner's syndrome (14/17), dysphagia (11/17), dysphonia (10/17), and ataxia of the limbs (10/17) or the trunk (15/17), whereas dysarthria (6/17) and hiccups were less common (3/17). None of the patients presented with new-onset (ipsilesional) hearing loss. Using a graded truncal ataxia rating system, severe (grade 3) truncal ataxia was noted in five patients, whereas moderate (grade 2) truncal ataxia was present in nine patients. Mild truncal ataxia (grade 1) was noted in a single patient only and two patients had no signs of truncal ataxia (grade 0).

**Table 1:** Epidemiologic and clinical findings in 17 acutely dizzy patients with MRI-confirmed unilateral lateral medulla syndrome (LMS).

**#**	Age^*^	**Diagnosis**	**Symptom-to-admission time (day)**	**Obvious neurologic signs**									
				**Ipsilesional facial hypesthesia**	**Contra-lesional body hypesthesia**	**Hiccups**	**Dysarthria/** **dysphonia**	**Dysphagia**	**Ipsi-lesional Horner syndrome**	**Limb ataxia**	**Truncal ataxia**	**Grading truncal ataxia^‡^**	**Ocular lateropulsion toward to lesioned side**
1	56–60	L LMS	3	+	–	–	+/+	+	+	+	+	2	+
2	46–50	R LMS	<1	+	+	+	–/–	–	–	+	–	0	–
3	66–70	L LMS	<1	+	–	–	–/–	–	–	+	+	3	+
4	36–40	L LMS	4	+	+	–	–/–	–	+	–	+	1	+
5	51–55	L LMS^†^	<1	–	+	–	–/–	–	+	–	+	2	+
6	41–45	L LMS^†^	<1	+	+	–	+/+	+	+	–	–	0	+
7	36–40	R LMS^†^	<1	+	+	–	±	+	+	+	+	3	+
8	56–60	R LMS^†^	<1	+	–	+	±	+	+	–	+	2	+
9	46–50	R LMS	2	+	+	–	–	–	+	+	+	3	+
10	66–70	R LMS	4	+	+	–	±	+	+	–	+	2	+
11	66–70	R LMS^†^	<1	+	–	–	+/+	–	+	+	+	2	+
12	76–80	R LMS^†^	<1	+	+	–	+/+	+	+	+	+	3	+
13	51–55	L LMS	16	+	+	–	–/–	+	+	+	+	2	–
14	76–80	L LMS	12	–	–	–	±	+	+	–	+	2	–
15	66–70	L LMS	1	–	+	–	+/–	+	+	–	+	2	–
16	86–90	L LMS	2	–	–	–	+/–	+	+	+	+	3	–
17	51–55	R LMS^†^	<1	–	–	+	–/–	+	–	+	+	2	+
All	59.4 ± 14.3 (avg ± 1 SD)	R:8, L:9		12/17 (70.6%)	10/17 (58.8%)	3/17 (17.6%)	6/17 (35.3%) and 10/17 (58.8%)	11/17 (64.7%)	14/17 (82.4%)	10/17 (58.8%)	15/17 (88.2%)	Gr 3: 5/17 Gr 2: 9/17 Gr 1: 1/17 Gr 0: 2/17	12/17 (70.6%)

*F, female; Gr: grade; L, left; LMS, lateral medullary stroke, M: male; R, right*.

* *In order to avoid indirectly identifiable patient data, age is shown in ranges only and gender has been removed*.

† *Initially false-negative MRI, diagnosed on follow-up MRI 3–4 days later*.

‡ *Grade 0: normal, able to stand on Tandem Romberg with the eyes open for at least 3 s; Grade 1: mild, unable to stand on Tandem Romberg with the eyes open for at least 3 s; Grade 2: moderate, unable to stand on Romberg test with the eyes open for at least for 3 s; Grade 3: severe, unable to stand or sit without support. This grading system was adapted from Moon and colleagues (12)*.

#### Subtle Ocular Motor Signs at the Bedside

The bedside horizontal head-impulse test was negative in 14 out of 15 patients (missing data in two patients), whereas it was positive ipsilesionally to the stroke in one patient. GEN was present in six out of 15 patients (missing data in two patients) and a skew deviation on the alternating cover test was found in four out of 15 patients (missing data in two patients). All patients had at least one component of H.I.N.T.S. (24) pointing to a central origin (INFARCT, 17/17), even though not all three components were checked in four patients. Specifically, patients #1 and 2 showed a normal bedside HIT even though GEN was not checked, and patients #11 and #16 had GEN whereas bedside HIT and test of skew were not performed. Ipsilesional ocular lateropulsion, as assessed by brief (3–5 s) eye closure as previously described by Kattah and colleagues (25), was noted in 12 out of 17 patients.

### Quantitative Vestibular and Ocular Motor Testing

#### Quantitative Assessment of the aVOR by Use of vHIT

**Figure 1:**
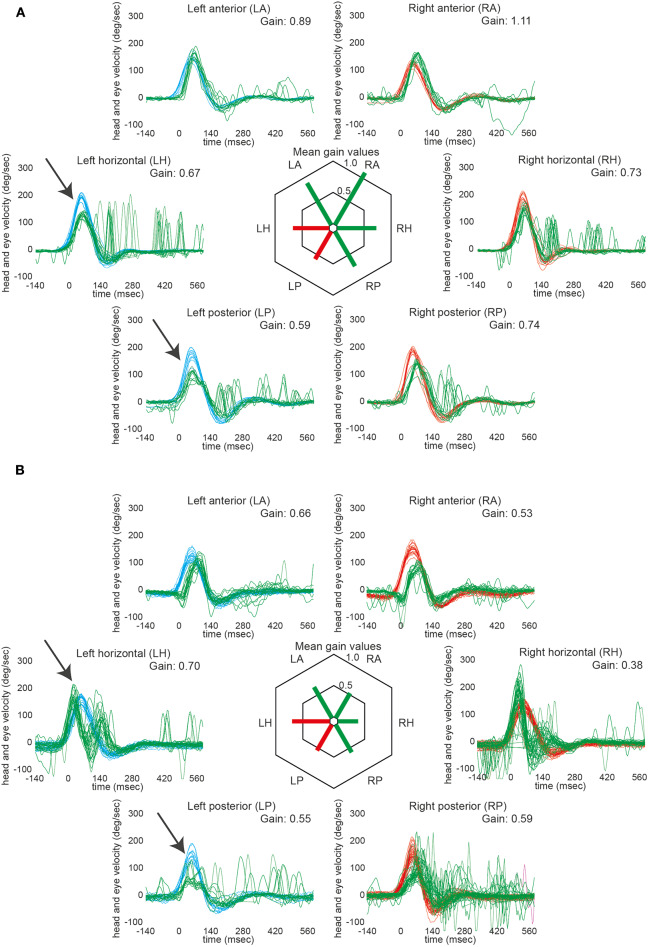
VHIT in those two patients that showed ipsilesionally impaired aVOR responses (#15 and 16). Note that both patients had ischemic lesions located at the rostral level of the dorsolateral medulla oblongata. In the left panel (**A**, #15), responses of the left horizontal and the left posterior semicircular canal were rated as impaired by the two reviewers (indicated by arrows). The mild reduction in gain of the right horizontal canal (being accompanied by catch-up saccades) was considered to be a compensatory down-regulation of the non-affected side. In the right panel (**B**, #16), again responses of the left horizontal and the left posterior semicircular canal were considered to be impaired by the two reviewers (indicated by arrows). Note that the eye traces are lagging the head traces in both anterior canals; thus, the calculated reduction in gain is most likely an artifact (this is also supported by the lack of catch-up saccades). In the right horizontal semicircular canal, the initial eye velocity traces are overshooting and then stop early (not showing consistent catch-up saccades); thus, the reduced gain (0.38) was considered an artifact. For the right posterior canal, the gain is slightly reduced and there are early catch-up saccades. This canal was rated as being overall slightly impaired due to the gain reduction and the presence of catch-up saccades. In both panels, eye velocity traces (in green) and head velocity traces (in red for assessing the right vestibular organ and in blue for assessing the left vestibular organ) are plotted against time for each SCC (20 trials per canal recorded). Note that eye velocity traces were inverted for better visualization and comparison with the head velocity traces. In the center of both panels, average gains are provided for all six semicircular canals. Whereas, canals with overall normal function were plotted in green, those with impaired function were illustrated in red.

Impaired responses on vHIT toward the side of the lesion were noted in 2 out of 17 patients (#15 and #16), involving the lateral and posterior canal in both cases (see Figure 1 and Table 2). These patients showed mild-to-moderate gain reductions accompanied by catch-up saccades in three out of four occasions. Note that in patient #15, the bedside horizontal HIT was rated abnormal ipsilesionally as well, whereas in patient #16 no bedside HIT was obtained. In two patients (#16 and 17), the contralesional posterior canal was rated as impaired with slight gain reduction and presence of catch-up saccades. False-low vHIT gains due to artifacts as delayed onset of the eye velocity trace (most likely due to goggle slippage) were noted in three patients (#14, 15, and 16). Noteworthy, these traces did not show significant catch-up saccades.

**Table 2:** Quantitative, video-based head-impulse testing in the 17 LMS patients.

**#**	Age^*^	**Diagnosis**	**Ipsilesional vHIT—overall rating (gain)**			**Contralesional vHIT—overall rating (gain)**		
			**HSC**	**ASC**	**PSC**	**HSC**	**ASC**	**PSC**
1	56–60	L LMS	Normal (1.01)	Normal (0.76)	Normal (0.78)	Normal (1.21)	Normal (0.81)	Normal (0.76)
2	46–50	R LMS	Normal (1.22)	Normal (0.93)	Normal (0.98)	Normal (1.19)	Normal (1.00)	Normal (0.86)
3	66–70	L LMS	Normal (1.01)	Normal (1.01)	Normal (0.82)	Normal (0.98)	Normal (1.09)	Normal (0.86)
4	36–40	L LMS	Normal (1.09)	Normal (0.75)	Normal (0.76)	Normal (1.16)	Normal (0.72)	Normal (0.80)
5	51–55	L LMS	Normal (1.12)	Normal (0.86)	NA	Normal (1.27)	NA	Normal (0.98)
6	41–45	L LMS	Normal (0.93)	Normal (0.74)	Normal (0.93)	Normal (0.96)	Normal (0.79)	Normal (0.81)
7	36–40	R LMS	Normal (1.19)	Normal (0.90)	Normal (0.93)	Normal (1.04)	Normal (1.11)	Normal (0.86)
8	56–60	R LMS	Normal (1.33)	Normal (0.80)	Normal (0.87)	Normal (1.16)	Normal (0.94)	Normal (0.74)
9	46–50	R LMS	Normal (1.18)	Normal (1.15)	Normal (0.99)	Normal (1.23)	Normal (0.97)	Normal (1.00)
10	66–70	R LMS	Normal (0.93)	Normal (0.64)	Normal (0.92)	Normal (0.98)	Normal (0.58)	Normal (0.76)
11	66–70	R LMS	Normal (1.20)	Normal (0.80)	Normal (0.80)	Normal (1.25)	Normal (0.92)	Normal (0.83)
12	76–80	R LMS	Normal (1.05)	Normal (1.00)	Normal (1.15)	Normal (0.95)	Normal (0.83)	Normal (0.85)
13	51–55	L LMS	Normal (1.11)	Normal (0.96)	Normal (0.67)	Normal (1.12)	Normal (0.87)	Normal (0.83)
14	76–80	L LMS	Normal (0.67)^†^	Normal (0.65)^†^	Normal (0.68)^†^	Normal (0.74)^†^	Normal (0.82)	Normal (0.70)
15	66–70	L LMS	Abnormal (0.67)	Normal (0.89)	Abnormal (0.59)	Normal (0.73)^‡^	Normal (1.11)	Normal (0.74)
16§	86–90	L LMS	Abnormal (0.70)	Normal (0.66)^†^	Abnormal (0.55)	Normal (0.38)^†^	Normal (0.53)^†^	Abnormal (0.59)
17	51–55	R LMS	Normal (1.01)	Normal (0.84)	Normal (0.88)	Normal (0.93)	Normal (1.10)	Abnormal (0.66)

*ASC, anterior semicircular canal; HSC, horizontal semicircular canal; LMS, lateral medullary stroke; PSC, posterior semicircular canal; vHIT, video-head-impulse test*.

* *In order to avoid indirectly identifiable patient data, age is shown in ranges only and gender has been removed*.

† *False-low vHIT-gain due to artifacts as delayed onset of the eye velocity trace (most likely due to goggle slippage). In addition, these traces did not show significant catch-up saccades*.

‡ *Mild gain reduction and overt catch-up saccades on the contralesional side, most likely reflecting compensatory down-regulation*.

Quantitative saccade analysis (OtosuiteV 4.0) was performed in all patients (see Table 3). Covert saccades were identified in a small fraction of trials only, averaging below 10%, with average CS latencies between 84 and 111 ms and with average CS peak velocities between 42 and 104°/s. Overt saccades were identified somewhat more frequently, with average fractions as high as 32 ± 34% for the ipsilesional horizontal SCC, but being clearly lower for the other SCCs. Average overt saccade latencies were between 314 and 368 ms and average OS peak velocities were around 90 to 130°/s.

**Table 3:** Quantitative analysis of catch-up saccades.

	**Ipsilesional side**			**Contralesional side**		
**Covert Saccades**	**HSC**	**ASC**	**PSC**	**HSC**	**ASC**	**PSC**
*% covert saccades*						
Range (%)	0–42	0–36	0–26	0–18^*^	0–38	0–31
Mean ± 1SD (%)	7 ± 11	6 ± 12	6 ± 8	9 ± 24	4 ± 10	3 ± 8
*CS latency (ms)*						
Mean ± 1 SD (%)	111 ± 31	88 ± 11	90 ± 11	95 ± 10	84 ± 9	91 ± 0
Patients w. CS (*n*)	9	3	6	8	4	2
*CS peak velocity (°/s)*						
Mean ± 1 SD (%)	98 ± 62	50 ± 31	52 ± 37	68 ± 37	42 ± 57	104 ± 24
Patients w. CS (*n*)	9	3	5	8	5	2
**Overt Saccades**	**HSC**	**ASC**	**HSC**	**ASC**	**HSC**	**ASC**
*% overt saccades*						
Range (%)	0–95	0–11	0–29	0–50	0–33	0–36
Mean ± 1 SD (%)	32 ± 34	1 ± 3	9 ± 11	14 ± 18	4 ± 10	5 ± 10
*OS latency (ms)*						
Mean ± 1 SD (%)	344 ± 86	368 ± 125	346 ± 111	319 ± 104	314 ± 118	325 ± 157
Patients w. OS (*n*)	14	3	10	12	5	5
*OS peak velocity (°/s)*						
Mean ± 1 SD (%)	121 ± 48	90 ± 16	130 ± 77	100 ± 55	90 ± 33	113 ± 85
Patients w. OS (*n*)	13	3	10	12	4	5

*CS, covert saccades; OS, overt saccades*.

* *In one patient (#12) the algorithm falsely detected a high gain as covert catch-up saccades, resulting in 100% covert saccades. This value was excluded from the analysis*.

#### SN and HSN in LMS

Video-oculography was obtained in 15 patients, demonstrating SN in 10 patients and significant HSN in 7 patients. Spontaneous horizontal nystagmus (range = 2.0–13.5°/s) was more often contraversive than ipsiversive (6 vs. 3) and one subject showed purely torsional nystagmus beating toward the side of the lesion. Accompanying spontaneous upbeat nystagmus (*n* = 4; range = 6.9–11.0°/s) or downbeat nystagmus (*n* = 1, 3.3°/s) was noted in five patients, whereas a torsional component was observed in two patients.

**Figure 2:**
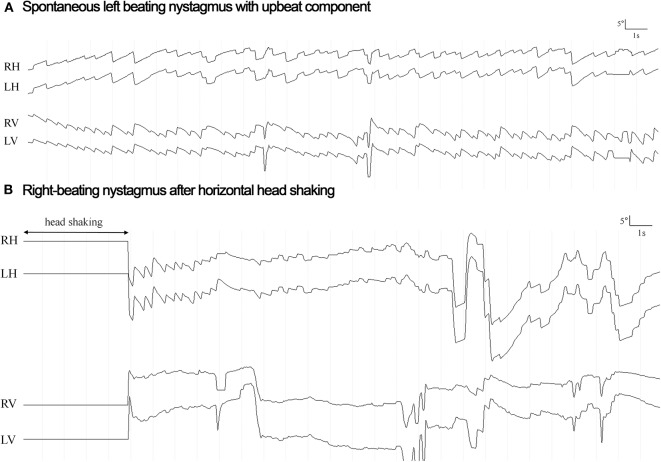
Horizontal eye position for the right eye (RH) and the left eye (LH) is plotted against time both at rest (panel **A**: spontaneous nystagmus) and after head shaking for 15 s (panel **B**: head shaking nystagmus) in a single patient (#8) with right lateral medullary infarction and normal responses on video-head-impulse testing. The direction of horizontal nystagmus is changed from left-beating (6.0°/s) to right-beating (21.4°/s) (toward the lesioned side) after head shaking, reflecting a central-type HSN response. Vertical eye position is also plotted both for the right (RV) and for the left (LV) eye.

HSN (according to the diagnostic criteria outlined in the *Materials and methods* section) was observed in seven out of 15 patients. While it was purely horizontal in six out of seven patients, it was purely vertical (down-beating) in one patient. The horizontal component was ipsilesional in all six patients presenting with horizontal HSN and was either reflecting a shift from contralesional SN to ipsilesional HSN (2/6), a significant increase in nystagmus velocity compared to the ipsilesional SN (2/6) or an ipsilesional HSN in the absence of a SN (2/6). In two patients (#7 and #8) the direction of the HSN changed from beating ipsilesionally to contralesionally over time (see Figure 2 for an example).

#### Horizontal GEN in LMS

Horizontal gaze-holding properties were assessed by VOG in 15 out of 17 patients in our study and deficits in gaze holding were identified in five patients. Compared to the results from bedside qualitative GEN testing, four patients demonstrated GEN in both tests, whereas one patient (#5) had GEN only on VOG, and in another patient (#8), GEN initially observed at the bedside was not seen on VOG obtained 3 days later (see Table 4 for details).

**Table 4:** Subtle ocular motor findings at the bedside and on VOG in 17 patients with MRI-confirmed unilateral LMS.

**#**	Age^*^	**Diagnosis**	**Subtle ocular motor findings at the bedside**			**Ocular motor findings on VOG**			**Either bedside or VOG**
			**Impaired bedside horizontal HIT**	**Bedside GEN**	**SD^†^**	**Spontaneous nystagmus (**°**/s)**	**HSN (**°**/s)^‡^**	**GEN on VOG**	**GEN**
1	56–60	L LMS	–	NA	L	–	**Ipsiles., LB (12.8)**	–	–
2	46–50	R LMS	–	NA	–	–	**Ipsiles., RB (4.7)**	–	–
3	66–70	L LMS	–	–	–	NA	NA	NA	–
4	36–40	L LMS	–	–	–	–	–	–	–
5	51–55	L LMS	–	–	–	Ipsiles., LB (2.0)	Ipsiles., LB (2.4), **DB (2.6)**	+	+
6	41–45	L LMS	–	–	–	–	Ipsiles., LB (1.4)	–	–
7	36–40	R LMS	–	–	–	Contrales., LB (6.4), UB (6.9), CCW (3.2)	**Ipsiles., first RB (5.6) then LB (10.0)**, UB (4.9), CCW (3.0) ^§^	–	–
8	56–60	R LMS	–	+	–	Contrales., LB (6.0), UB (10.2)	**Ipsiles., first RB (21.4) then LB (5.0) ^§^**	–	+
9	46–50	R LMS	–	+	–	Ipsiles., RB (3.5), UB (10.8)	**Ipsiles., RB (9.0)**	+	+
10	66–70	R LMS	–	–	–	Contrales., LB (13.2), UB (11.0)	Contrales., LB (9.2), UB (8.1)	–	–
11	66–70	R LMS	NA	+	NA	Ipsiles., RB (2.7)	**Ipsiles., RB (6.4)**, DB (1.6)	+	+
12	76–80	R LMS	–	–	R	Ipsiles., CCW (3.5)	–	–	–
13	51–55	L LMS	–	–	L	Contrales., RB (4.0)	Contrales., RB (3.0)	–	–
14	76–80	L LMS	–	+	L	–	Ipsiles., LB (2.1)	+	+
15	66–70	L LMS	L	+	–	NA	NA	NA	+
16	86–90	L LMS	NA	+	NA	Contrales., RB (2.8), DB (3.3)	Contrales., RB (2.5), DB (3.2)	+	+
17	51–55	R LMS	–	–	–	Contrales., LB (4.7)	–	–	–
**All**	**59.4 ± 14.3 (avg±1 SD)**	**R:8, L:9**	**1/15 (7%)**	**6/15 (40%)**	**4/15 (27%)**	**Horizontal contrales.: 6/15 (40%)** **Horizontal ipsiles.: 3/15 (20%)** **Purely torsional: 1/15 (7%)** **None: 5/15 (33%)**	**Ipsiles.: 6/15 (40%)** **vertical: 1/15 (7%)** **Non-significant or none: 8/15 (53%)**	**5/15 (33.3%)**	**7/17 (41.2%)**

*contrales., contralesional; CCW, counterclockwise; CW, clockwise; GEN, gaze-evoked nystagmus; HSN, head-shaking nystagmus; ipsiles., ipsilesional; L, left; LB, left-beating; LMS, lateral medullary stroke, R, right; RB, right-beating; SD, skew deviation; VOG: video-oculography; UB, up-beating*.

* *In order to avoid indirectly identifiable patient data, age is shown in ranges only and gender has been removed*.

† *The indicated eye (L or R) refers to the hypotropic eye*.

‡ *For HSN, those measurements that met diagnostic criteria for significant HSN as defined in the methods section are in bold*.

§ *In two patients (#7 and #8), the direction of the head-shaking nystagmus changed from beating ipsilesionally to contralesionally over time*.

### Location and Extent of Ischemic Lesions on Brain MRI

**Figure 3:**
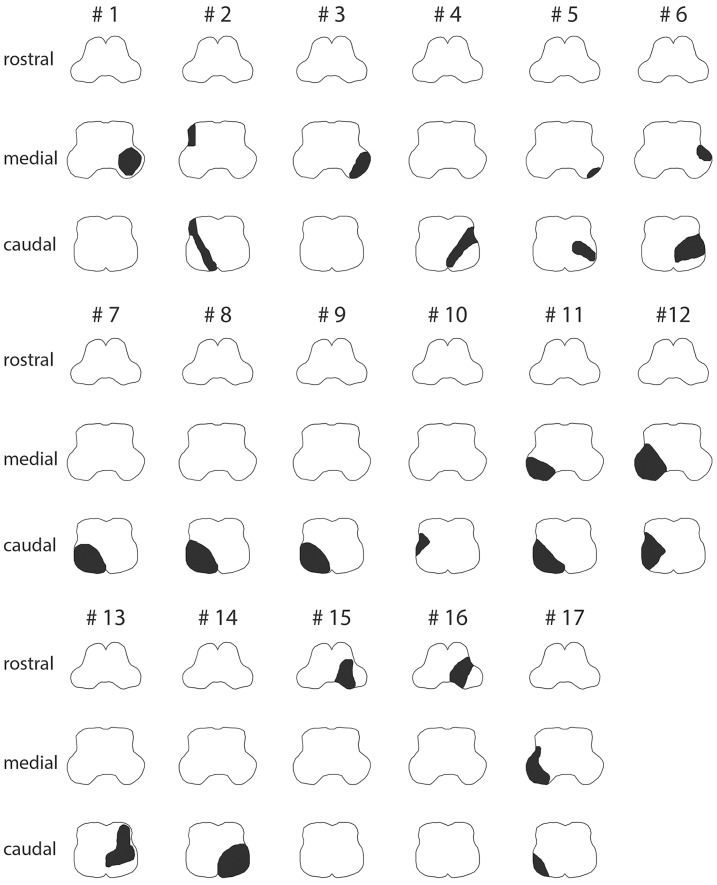
Graphical illustration of the extent of ischemic lesions (in black) at the caudal, middle, and rostral level of the medulla oblongata in all 17 patients as identified on MR imaging (see Figure S1 for corresponding MR-images).

The majority of LMS were located at the level of the caudal (*n* = 7), the caudal and middle (*n* = 6), or the middle (*n* = 2) medulla oblongata, whereas only two patients (#15 and #16) presented with lesions of the rostral medulla oblongata (Figure 3, Figure S1 and Table 5). Combined involvement of the lateral and posterior vascular territory was seen most often (*n* = 13), while restriction to the lateral (*n* = 2), or posterior (*n* = 1) territory was less common. Only one patient showed ischemia of the anterolateral (and lateral) territory.

**Table 5:** MRI findings in 17 patients with unilateral LMS.

**#**	Age^*^	**Diagnosis**	**Medullary lesion level^†^**	**Vascular supply regions affected^‡^**	**MRI-based lesion rating [±]**							
					**SVN**	**LVN**	**MVN**	**IVN**	**NP**	**ICP**	**CBL**	**ION**
1	56–60	L LMS	Middle	Lateral and posterior	–	–	+	+	+	+	–	–
2	46–50	R LMS	Middle and caudal	Lateral and posterior	–	–	+	–	+	–	–	+
3	66–70	L LMS	Middle	Posterior	–	–	+	+	+	–	–	–
4	36–40	L LMS	Caudal	Lateral and posterior	–	–	–	–	–	–	–	+
5	51–55	L LMS	Middle and caudal	Lateral and posterior	–	–	–	+	–	–	–	+
6	41–45	L LMS	Middle and caudal	Lateral	–	–	–	–	–	–	–	–
7	36–40	R LMS	Caudal	Lateral and posterior	–	–	+	+	+	+	–	–
8	56–60	R LMS	Caudal	Lateral and posterior	–	–	+	+	+	–	–	+
9	46–50	R LMS	Caudal	Lateral and posterior	–	–	+	+	+	+	–	–
10	66–70	R LMS	Caudal	Lateral	–	–	–	–	–	–	–	+
11	66–70	R LMS	Middle and caudal	Lateral and posterior	–	–	+	+	+	–	–	–
12	76–80	R LMS	Middle and caudal	Lateral and posterior	–	–	+	+	–	–	–	+
13	51–55	L LMS	Caudal	Lateral and anterolateral	–	–	–	–	–	–	–	+
14	76–80	L LMS	Caudal	Lateral and posterior	–	–	+	+	+	–	–	–
15	66–70	L LMS	Rostral	Lateral and posterior	–	–	+	–	+	–	–	–
16^§^	86–90	L LMS	Rostral	Lateral and posterior	–	–	+	–	+	–	+ (Fl)	–
17	51–55	R LMS	Middle and caudal	Lateral and posterior	–	–	–	+	+	–	–	–
All			Rostral = 2 Middle = 2 Middle and caudal = 6 Caudal = 7	Anterolateral and lateral = 1 Lateral = 2 Lateral and posterior = 13 Posterior = 1	0/17 (0%)	0/17 (0%)	11/17 (65%)	10/17 (59%)	11/17 (65%)	3/17 (18%)	1/17 (6%)	7/17 (41%)

*CBL, cerebellum; ICP, inferior cerebellar peduncle; ION, inferior olivary nucleus; IVN, inferior vestibular nucleus; LMS, lateral medullary syndrome; NP, nucleus prepositus*.

* *In order to avoid indirectly identifiable patient data, age is shown in ranges only and gender has been removed*.

† *Lesion levels as defined in the methods section based on the publications by Bradley (21) and Kim and colleagues (2)*.

‡ *Four penetrating arteries provide vascular supply to the medulla oblongata; thus, four regions are distinguished: anteromedial, anterolateral, lateral, and posterior (22, 23)*.

§ *In this patient, the MRI demonstrated involvement of the left cerebellar flocculus and of the dorsal pons (left side) as well*.

MRI-confirmed lesions in our 17 patients involved parts of the VN in 13/17, with the medial VN being affected most often (11/17), followed by the inferior VN (10/17), whereas none of the patients showed involvement of the superior VN or the lateral VN. Noteworthy, it was usually the most caudal portion of the medial VN and the inferior VN that was affected. Other structures that were affected as well included the NP (11/17), the ION (7/17), and the ICP (3/17). In one patient (#16), the lesion was reaching further rostral (including the area of the VN in the lower pons), and this patient also demonstrated acute ischemic stroke of the left cerebellar flocculus. In the other 16 patients, no acute ischemic cerebellar lesions were noted.

Initial MRI (including DWI) was false negative in seven patients. All these patients received initial MR imaging within 24 h of symptom onset and had stroke confirmed on follow-up imaging 3–4 days later. Noteworthy, the rate of false-negative MRI in LMS was 70% when considering only those patients that received initial MR imaging within 24 h of symptom onset.

## Discussion

Ischemic lesions involving the (dorso-) lateral medulla result in a characteristic clinical picture known as Wallenberg syndrome with various brainstem signs. Most commonly, these patients report vertigo or dizziness along with gait imbalance, nausea, and nystagmus (3, 4), thus meeting diagnostic criteria for an acute vestibular syndrome (AVS) (26, 27). The aim of this study was to characterize the vestibular and ocular motor properties in these patients with a special focus on the integrity of the VN, as they may be damaged as well in patients with LMS. Thus, we predicted an impaired aVOR in those patients with substantial involvement of the VN, whereas the aVOR will be spared in the others.

### Why the aVOR Remained Intact in Most of our LMS Patients

Our patients presented with both vestibular symptoms [dizziness (in all 17 patients), and SN and/or HSN (in 13/15 patients tested)] and truncal ataxia (15/17), thus pointing to an involvement of primarily the caudal portions of the lateral medulla (28). At the same time, typical findings of more rostral (middle section) lateral medulla lesions as facial palsy (none) or dysarthria (6/17) were less common. Using both bedside and quantitative vHIT, we noted minor ipsilesional deficits in the aVOR in only two patients (and additional contralesional deficits in two patients). On brain MRI, ischemic medullary lesions were restricted to the caudal or middle level in all but two patients that showed lesions at the rostral level. Whereas, the superior and lateral VN were spared in all patients, involvement of the medial and/or the inferior VN was found in 13 patients (with the other four patients presenting with purely lateral or anterolateral medullary strokes). Thus, in medullary stroke patients with prominent vestibular symptoms, more emphasis should be put on a dorsolateral location of the lesion. Noteworthy, ipsilesional deficits in the aVOR on vHIT were noted only in those two patients with lesions in the lateral and posterior parts of the rostral medulla oblongata. The finding that the aVOR remained functionally intact in all but two of our LMS patients can be explained by the relative location of the ischemic lesions to the VN. The VN complex is centered around the dorsolateral aspects of the pontomedullary junction. The larger two of its four nuclei, i.e., the medial and the inferior VN, extend cranially into the caudal pons and reach down to the floor of the fourth ventricle (11), whereas the smaller two nuclei, i.e., the superior and lateral VN, are located in the dorsolateral pons above the inferior and the medial VN. Thus, in patients with lesions restricted to the caudal or middle level of the dorsolateral aspects of the medulla, only the most caudal parts of the medial and inferior VN will be damaged, sparing most of these two nuclei. Specifically, this includes sparing of the vestibulo-ocular neurons, as they are concentrated in the rostral third of the medial VN (29), providing a potential explanation why the aVOR remained functionally intact in these patients.

### Subtle Ocular Motor Signs in LMS

The bedside neuro-otologic evaluation supported a central-type AVS in all 17 patients. Specifically, the H.I.N.T.S. had a 100% sensitivity for identifying a central cause, and additional nystagmus patterns pointing to a central cause as vertical/torsional SN and ipsilesional or perverted HSN were found in 10 out of 15 patients. The fraction of LMS patients presenting with GEN (40% at the bedside) was comparable to the numbers reported from more heterogeneous cAVS populations, ranging between 13 and 57% (30–33). Ocular lateropulsion toward the lesioned side was found to be frequent in patients with LMS, being present in 71% in our case series.

#### SN Patterns in Lateral Medullary Stroke

The SN in LMS is usually caused either by acute lesions at the root entry zone of the vestibulocochlear nerve, within the VN or along the vestibulo-cerebellar projections. The nystagmus usually beats to the intact side, but may also be directed to the side of the infarction (34). According to an analysis of the nystagmus axis in LMS patients, the nystagmus pattern can be explained by an ipsilateral lesion of the central semicircular canal pathways of the anterior and horizontal semicircular canals (34).

In cases with spontaneous horizontal nystagmus (present in 9 out of our 17 patients), the direction of nystagmus was contraversive more often than ipsiversive (67 vs. 33%). Former reports regarding the beating direction of horizontal nystagmus in LMS showed that caudal lesions result in contralesional horizontal SN and lesions within the middle and rostral portions are associated with ipsilesional SN both in humans (14, 34–37) and in non-human primates (38). In our cases series, seven out of nine patients with contralesional horizontal SN had LMS restricted to the caudal medulla, whereas in the other two patients, lesions either were located at both the caudal and middle level of the medulla (#17) or were restricted to the rostral level (#16). Likewise, in two of three patients with ipsilesional horizontal SN, the middle level of the medulla was damaged as well (along with the caudal level), whereas in the third patient, the stroke was restricted to the caudal medulla. A vertical component was found in five patients and was usually up-beating (80%), being consistent with findings from previous publications (14, 35).

Previously, lesions of the nucleus prepositus (NP) and the medial VN have been linked to mostly horizontal nystagmus beating away from the affected side (39). The NP is located medially to the medial VN (at about the same rostro-caudal level) (40) and was likely damaged in those 11 patients with their stroke extending into the dorsal and medial parts of the medulla. Contralesional SN, however, was observed in 4 of these 11 patients with NP lesions only, whereas 2 showed ipsilesional SN and 5 showed no SN on VOG. Focusing on those six patients that presented with contralesional SN, four had lesions extending into the area of the NP, whereas two patients had more lateral and anterior lesions likely sparing the NP. These observations emphasize that various central structures may cause horizontal SN with varying beating directions in LMS.

Horizontal SN was accompanied by a vertical and/or torsional component in seven patients, whereas only one patient presented with a purely torsional nystagmus. While horizontal SN does not allow a distinction between a peripheral and a central pathology, vertical and/or torsional SN is indicative of a central cause (41), further narrowing the differential diagnosis in the evaluation of such patients.

#### Gaze-Holding Deficits in Lateral Medullary Stroke

Deficient eccentric gaze holding, resulting in a horizontal GEN, was noted in 40% of our patients when tested at the bedside on the ED. Horizontal gaze holding is provided by a brainstem horizontal neural velocity-to-position integrator (42, 43) that is located in the NP and in the medial VN (39). Robinson hypothesized that this neural velocity-to-position integrator is inherently “leaky” and thus requires cerebellar input to calibrate its output precisely in proportion to eye position (42). In our case series, 6 out of 11 patients (55%) with suspected lesions of the NP demonstrated horizontal GEN either at the bedside or on VOG. Thus, our numbers are somewhat lower than those reported in the literature, describing high rates of GEN (72%) in patients with infarctions involving the NP (44).

### Head-Shaking Nystagmus Patterns in Lateral Medullary Stroke

In our case series, significant head-shaking-induced nystagmus was observed in seven patients and was beating toward the lesioned side in all six patients with horizontal HSN, whereas another patient presented with downbeat nystagmus after head shaking. Thus, all patients demonstrating significant HSN in our study had a central-type pattern of HSN, either pointing toward the lesioned side (6/7) or meeting criteria for perverted nystagmus with vertical or torsional nystagmus induced by horizontal head shaking. Previously, HSN in LMS has been reported to beat ipsilesionally (14, 36), being consistent with our observations. Choi and colleagues proposed unilaterally impaired nodulo-uvular inhibition of the velocity storage resulting from lesions of the caudal or middle portions of the VN to explain the occurrence of ipsiversive HSN (14). Thus, with lesions centered around the more caudal parts of the medulla in most of our patients, the ipsilesionally directed HSN can be well-explained.

### Head-Impulse Testing (Either Bedside or Quantitative) in Lateral Medullary Stroke

In our case series of LMS patients presenting with a main complaint of acute-onset and persistent dizziness or vertigo, ipsilesional deficits in the aVOR as assessed both by bedside horizontal HIT and by the vHIT were infrequent. Specifically, ipsilesionally reduced gains and/or the occurrence of catch-up saccades on vHIT were noted in two patients only (12%). Furthermore, these deficits were mild and affected only selected semicircular canals (with anterior canal sparing). At the bedside, the HIT was ipsilesionally impaired in one of these two patients, while it was not assessed at the bedside in the other patient. This matches the MR-based ratings that showed minor involvement of the medial and the inferior VN only and no involvement of the superior and the lateral VN, i.e., leaving the critical structures for the aVOR mostly spared. At the bedside, the horizontal HIT was reported to be usually normal in LMS (24, 32, 45); exceptions included lesions involving the dorsolateral portions of the rostral medulla [with an abnormal HIT in one out of two patients in one study (45)]. Likewise, using bithermal caloric irrigation, Choi and colleagues reported ipsilateral (*n* = 1) or contralateral (*n* = 1) canal paresis only in 2 out of 15 cases with LMS involving the caudal and middle portions of the medulla (14). However, due to ocular lateropulsion, which is a frequent finding in LMS (9), the value of the bedside HIT (which is based on the visual detection of overt catch-up saccades, whenever the aVOR is deficient) in these patients is limited. Specifically, when performing the bedside HIT toward the lesioned side, elicited compensatory saccadic eye movements may be hypometric due to saccadic lateropulsion and thus the bedside HIT may be interpreted as false abnormal. We did not quantify saccadic lateropulsion in our patients; however, this should be evaluated in future studies on LMS. Noteworthy, as for the clinical interpretation, other central signs (i.e., the H.I.N.T.S. plus) will be assessed, and these patients will likely still be correctly identified as having a central AVS at the bedside.

Likewise, when assessing the vHIT, occurrence of compensatory overt catch-up saccades should be interpreted with caution. The quantitative saccade analysis performed identified overt catch-up saccades in 32% of vHIT traces on average (±34%, 1 SD) when testing the aVOR for the ipsilesional horizontal SCC (which triggers compensatory saccades away from the lesioned side). These catch-up saccades, however, may be related to ocular lateropulsion and not to a deficient aVOR. Noteworthy, in all individual vHIT traces rated as overall abnormal, the aVOR gain was reduced as well, thus making it unlikely that we rated aVOR responses as false abnormal due to ocular lateropulsion. Thus, presence of excessive catch-up saccades in vHIT in AVS patients should prompt search of saccadic abnormalities as saccadic lateropulsion and consideration of central (lateral medullary) lesions.

Thus, in LMS patients with persistent peripheral vestibular signs, a more rostral extension of the stroke (e.g., as combined PICA and AICA stroke or brainstem and cerebellar stroke with brainstem compression) must be considered as emphasized by Kattah and colleagues (46). Noteworthy, anterior canal sparing was also noted in two patients with unilateral ischemic lesions restricted to the medial VN or the medial and the inferior VN when assessing the aVOR quantitatively (47).

Considering the vestibular signs and symptoms observed—including vertigo and dizziness, skew deviation, and truncal ataxia—lesions along the central vestibular pathways (48, 49), such as damage of the vestibulo-cerebellar projections as, e.g., within the ICP (as observed in three patients in our case series), may be considered (3, 5). In contrast, in the dorsal medullary syndrome, abnormal bedside HIT was reported in 10 out of 18 patients, but is ipsilesionally impaired in 4 and contralesionally impaired in 6 patients. This may also explain why we noted contralesionally impaired aVOR responses on vHIT in two patients. A significant canal paresis (>25%) on caloric irrigation ipsilesionally was previously noted in 4 out of 15 patients (28).

With the pattern of vHIT abnormalities observed in our LMS patients (ipsilesional impairment of the lateral and posterior SCC), formally testing the vHIT in all three canals in AVS patients with uncertain localization may be helpful. Specifically, the combination of decreased aVOR gains in the ipsilesional horizontal (MVN) and posterior canal (IVN) differentiates it from a vestibular neuritis with decreased aVOR gains of the ipsilesional horizontal and anterior canal. Furthermore, a decreased contralesional horizontal gain may point to a central dorsal medulla localization.

### Limitations

This retrospective consecutive case series from a prospective stroke registry has several limitations. This includes a moderate sample size with a majority of LMS patients presenting with lesions at the caudal and/or middle medullary level. While this may represent the true distribution of LMS, the high dropout rate of eligible patients (69%) due to various causes may result in a selection bias and lesions of the rostral medulla may be under-represented in our case series, e.g., due to more intense nausea and vomiting in those cases with significant damage to the (medial) VN. While ideal slice thickness for DWI sequences is 3 mm, we used somewhat thicker slices (4 mm) in our study, which may have increased the risk for missing small diffusion restrictions. Furthermore, no dedicated (volumetric) MR imaging was available when assessing the extent of the medullary lesions, thus being potentially biased by the experience and preference of the reviewer. Also, the delay of testing after symptom onset showed variability. While the majority of patients presented to the emergency department within 24 h, two patients could be assessed only more than 10 days after symptom onset (12 and 16 days, respectively). Thus, some of the patients' symptoms may have already disappeared.

We observed high aVOR gains (i.e., being 1.2 or larger) in 5 of our 17 patients, which most likely is related to slippage of the vHIT goggles while applying the head impulses. Risk of goggle slippage depends on the shape of the patient's face and thus how well the google fits. In our patient population (recruited in South Korea), fit of the vHIT goggles may be imperfect and thus, this may have increased the likelihood of slippage. Alternatively, errors in the calibration procedure may have also resulted in false-high gains (17). However, with a patchy pattern of high aVOR gains in our patients, wrong calibration seems an unlikely cause of abnormally high aVOR gains in our study. Other vHIT-related artifacts previously reported include eyelid interference with the pupil, blinks, and head overshooting (17, 50). We identified head overshooting in one of these patients (patient #9). Overall, in those patients with clearly abnormal vHIT responses (patients #15–17), no high aVOR gains were observed in single SCCs. Thus, we do not think that abnormally high aVOR gains have significantly affected our test results or our interpretation of the study's findings.

## Conclusions

In patients with LMS, ipsilesional deficits in the aVOR as assessed by the (quantitative) head-impulse test are infrequent and—if present—usually mild and restricted to selected semicircular canals. This observation is consistent with the extent of the medullary lesions found on brain MRI, showing minor or no involvement of the VN complex ipsilesionally. At the same time, the bedside assessment for subtle ocular motor signs (24) had a high sensitivity for detecting central lesions. A central cause was also supported by the presence of vertical/torsional SN and a central-type head-shaking-induced nystagmus, further emphasizing the usefulness of assessing ocular motor properties in these patients.

## Data Availability Statement

The datasets generated for this study are available on request to the corresponding author.

## Ethics Statement

The studies involving human participants were reviewed and approved by Institutional Review Board of the Chonnam National University Hospital (Gwangju, South Korea). The patients/participants provided their written informed consent to participate in this study.

## Author Contributions

S-HL and AT: material preparation, data collection and analysis, and writing of the first draft of the manuscript. J-MK: data collection and analysis. BS: review of the MR images. All authors read, revised, and approved the final version of the manuscript.

## Conflict of Interest

The authors declare that the research was conducted in the absence of any commercial or financial relationships that could be construed as a potential conflict of interest.
