# Supplementary material for: Vestibular and Ocular Motor Properties in Lateral Medullary Stroke Critically Depend on the Level of the Medullary Lesion
Source: Front Neurol. 2020 Jun 5;11:390. doi: 10.3389/fneur.2020.00390 (PMC7325917; doi:10.3389/fneur.2020.00390)
Supplement: Supplementary file 1 [file Data_Sheet_1.pdf]

Supplementary material

Figure S1:

Axial diffusion-weighted (#2-12, #14-17) or T2-weighted (#1 and 13) MR-images at the level of the caudal (left column), middle (middle column) and rostral (right column) medulla are shown.

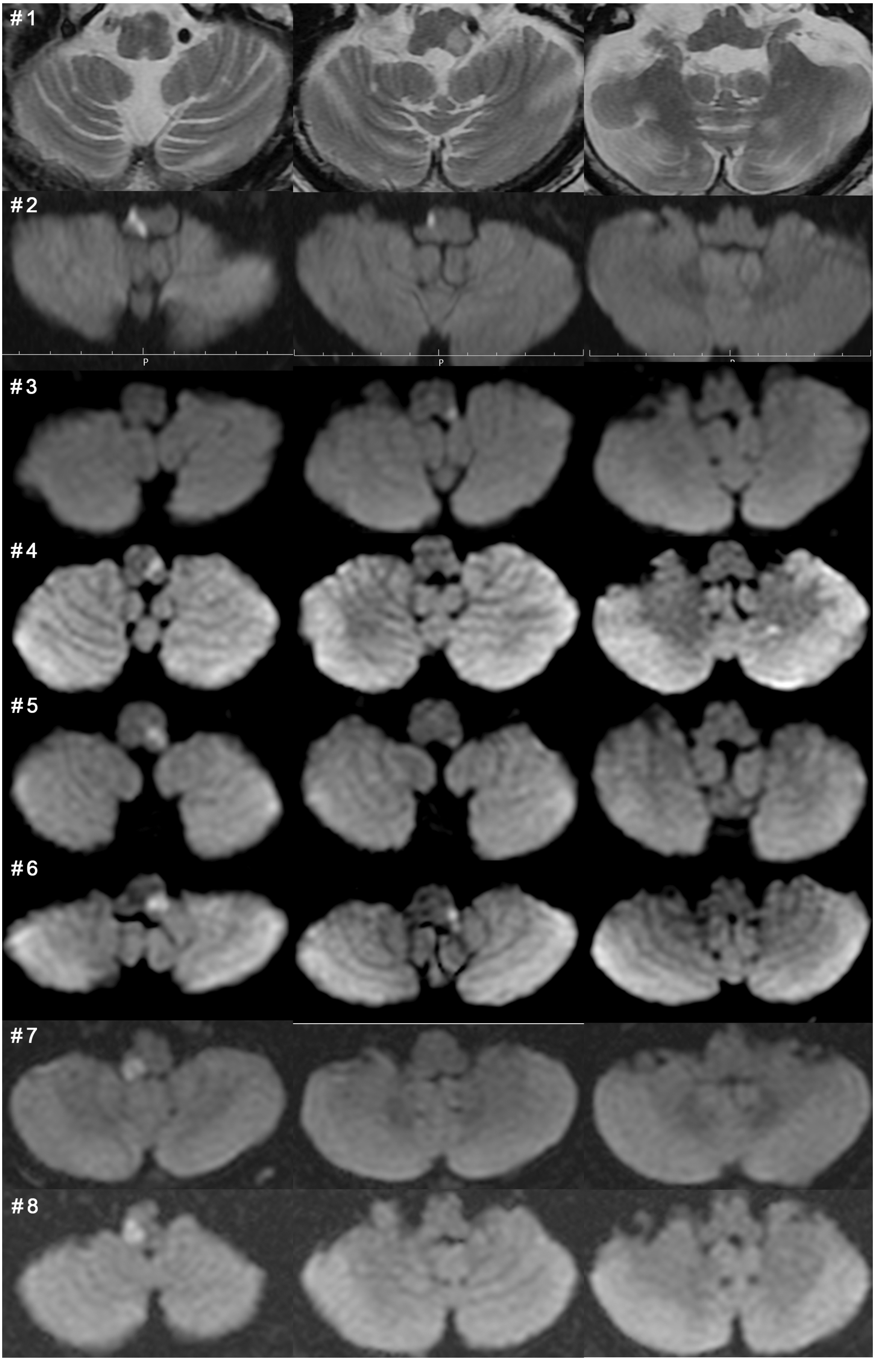
#1
#2
#3
#4
#5
#6
#7
#8

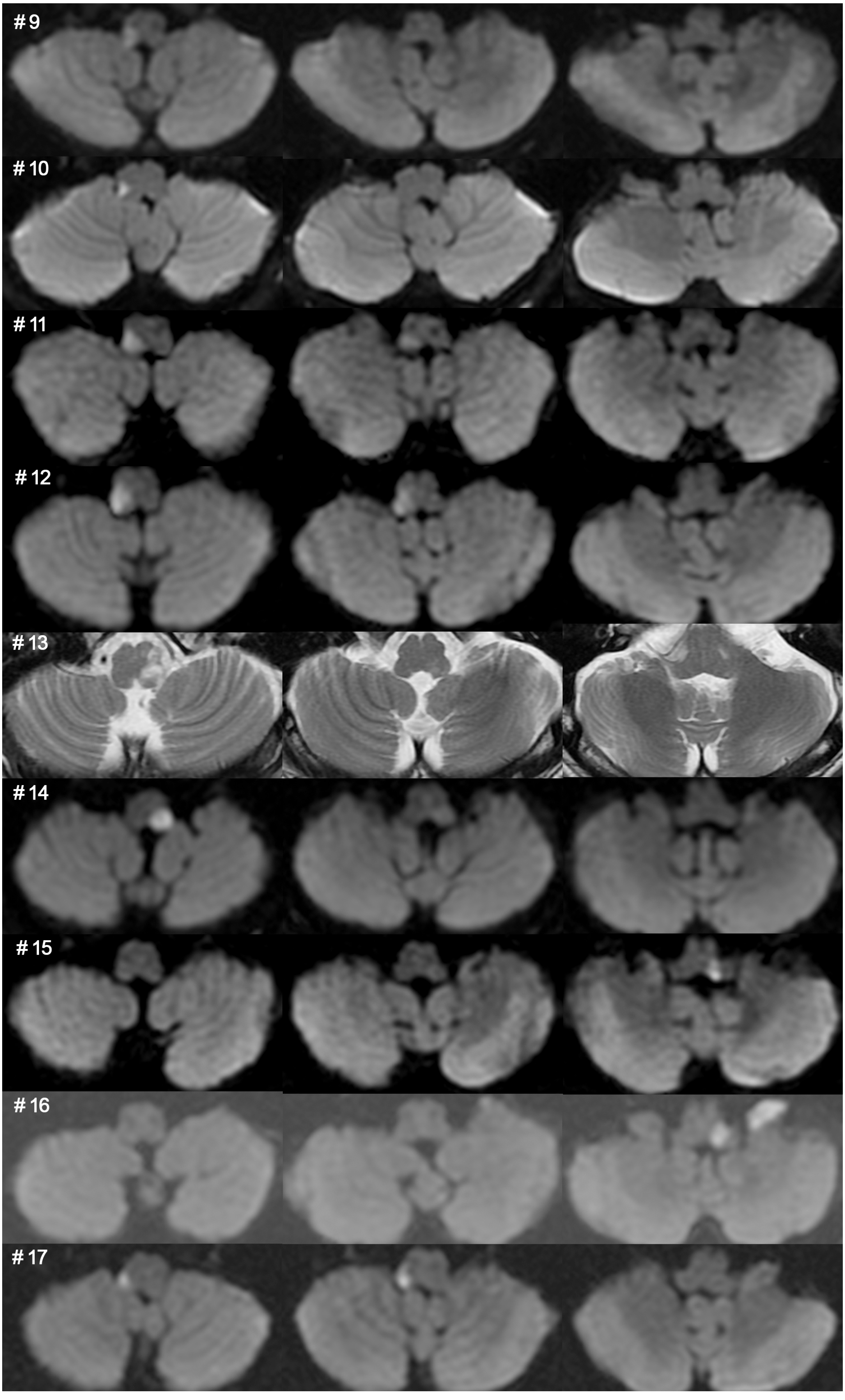

9
10
11
12
13
14
15
16
17
